# Bare parts in the Galliformes: the evolution of a multifunctional structure

**DOI:** 10.1098/rsos.231695

**Published:** 2024-01-10

**Authors:** Min Zhao, Sarah M. Kurtis, Ellen A. Humbel, Emily V. Griffith, Tong Liu, Edward L. Braun, Richard Buchholz, Rebecca T. Kimball

**Affiliations:** ^1^ Department of Biology, University of Florida, Gainesville, FL 32611, USA; ^2^ Department of Biodiversity, Earth, and Environmental Sciences, Drexel University, Philadelphia, PA 19104, USA; ^3^ College of Life Science, Jilin Agricultural University, Jilin, People's Republic of China; ^4^ Department of Biology, University of Mississippi, University, MS 38677, USA

**Keywords:** landfowl, fleshy structure, sexual selection, natural selection, phylogenetic comparative analysis

## Abstract

A morphological trait can have multiple functions shaped by varying selective forces. Bare parts in birds, such as wattles, casques and combs, are known to function in both signalling and thermoregulation. Studies have demonstrated such structures are targets of sexual selection via female choice in several species of Galliformes (junglefowl, turkeys and grouse), though other studies have shown some role in thermoregulation (guineafowl). Here, we tested fundamental hypotheses regarding the evolution and maintenance of bare parts in Galliformes. Using a phylogeny that included nearly 90% of species in the order, we evaluated the role of both sexual and natural selection in shaping the function of bare parts across different clades. We found a combination of both environmental and putative sexually selected traits strongly predicted the variation of bare parts for both males and females across Galliformes. When the analysis is restricted to the largest family, Phasianidae (pheasants, junglefowl and allies), sexually selected traits were the primary predictors of bare parts. Our results suggest that bare parts are important for both thermoregulation and sexual signalling across Galliformes but are primarily under strong sexual selection within the Phasianidae.

## 1. Introduction

Many species exhibit bright colours or specialized structures that are thought to be involved in signalling. Traits that vary seasonally or are primarily exhibited in males are often thought to have evolved via sexual selection. In birds, much research has been dedicated to understanding the role of avian plumage coloration and elaboration as it relates to courtship and female mate choice. However, bare parts, i.e. body parts that are not covered by feathers, such as eye rings, wattles, caruncles, gular sacs, casques, combs and horns, are also broadly distributed among birds [1–4]. Like plumage, these featherless regions and structures can also be strikingly coloured, sexually dimorphic, and vary temporally (sometimes changing over seasons or even minutes), suggesting they could also function as key visual signals in many avian species (e.g. [5,6]; reviewed by [4]).

Unlike plumage, which is typically replaced only once or twice each year, the appearance of bare parts can reflect short-term changes in physiology. For example, the colour of these regions is often dependent upon androgens (e.g. [7]) and so will change in response to hormonal changes within an individual. Since androgen levels may decrease in individuals that are ill or in poor condition [8], the appearance of bare parts can provide real-time information about an individual's status, which would make bare parts reliable, honest signals of current individual quality, and therefore likely targets of sexual selection [9–11]. Unsurprisingly, several studies have found support for the use of bare parts in signalling in birds, such as cassowaries [12], Malagasy asities [6], caracaras [1] and galliform birds like Wild Turkey *Meleagris gallopavo* [13], Red Junglefowl *Gallus gallus* [5,14] and Black Grouse *Tetrao tetrix* [15].

Bare parts may also be under natural selection. These regions lack insulation, and thus can be a greater source of heat loss than other body parts (e.g. [16,17]). Although most birds have featherless skin on their bill, tarsus, and toes, thermoregulation may be particularly important for species that have specialized bare parts, such as wattles, eye rings, or featherless heads, in that these species have more surface area for heat exchange. Cold environments could impose heavy costs on bare parts, which may be why some arctic birds have feathered legs and feet (e.g. ptarmigans [18] and Snowy Owl *Bubo scandiacus* [19]), while in very hot environments, such regions could be selected for to dissipate heat (e.g. [20–22]). Given potential energetic costs of generating and maintaining these bare structures [13,23], it is unlikely that purely neutral processes drive their evolution.

**Figure 1.**
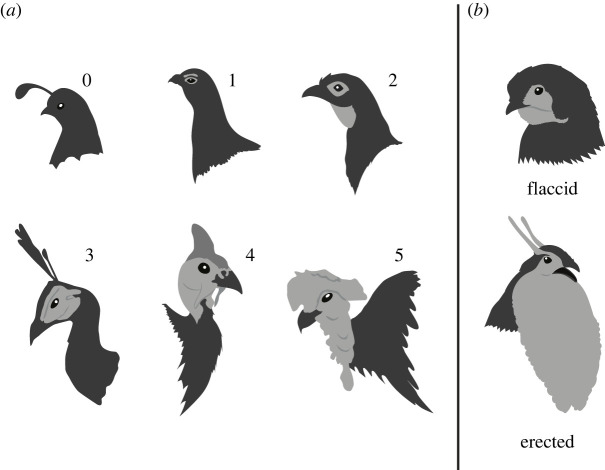
Examples of bare parts in Galliformes. (*a*) Examples of bare parts ranging from score zero to five for bare part size: 0) Gambel's Quail *Callipepla gambelii*; 1) male Willow Ptarmigan *Lagopus lagopus*; 2) Red-faced Guan *Penelope dabbenei*; 3) male Green Peafowl *Pavo muticus*; 4) Helmeted Guineafowl *Numida meleagris*; and 5) Wattled Brushturkey *Aepypodius arfakianus*. (*b*) Example of 4X bare part size change during active display in male Temminck's Tragopan *Tragopan temminckii*. Drawings by E.A.H.

The avian order Galliformes (chickens, turkeys, quail, and other landfowl) contains many species that exhibit specific featherless structures ([4,24]; figure 1). This includes the well-studied combs of Red Junglefowl [5,25,26] and the snoods of Wild Turkeys [13,27], both of which are involved in intra- and intersexual selection. However, bare parts in Galliformes have also been demonstrated to be important for heat dissipation [21,22]. In some species within the Phasianidae, males can rapidly enlarge some bare parts using air, fluids or muscle changes (figure 1*b*; see also [28–31]), which may allow males to both have a large signal for display but also to minimize heat loss at other times as they mostly occur in the temperate or even subarctic zone. The earliest diverging clades within Galliformes (i.e. Megapodiidae, Cracidae and Numididae) contain species that primarily inhabit tropical regions and where bare parts, if present, are often similar in size between the sexes. Thus, while much research has focused on the role of these traits in sexual selection, this suggests a potential role of climate in the evolution and/or maintenance of specialized bare parts instead of, or in addition to, sexual selection [32].

**Figure 2.**
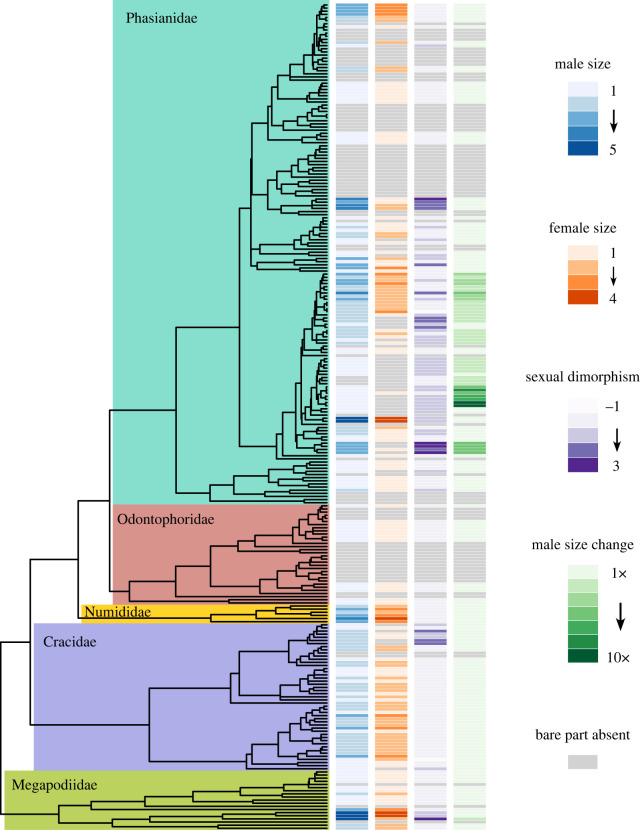
Phylogeny of Galliformes [33] with bars next to tree indicating scores for bare part size of both sexes, sexual size dimorphism and male size change. Grey bars indicate absence of bare part.

Here, we evaluated the evolution and maintenance of specialized bare parts in a phylogenetic framework within Galliformes, where these traits can show substantial variation (figure 2; electronic supplementary material, figure S1). Bare parts may be under both sexual and natural selection, may be driven by different selective pressures in different groups, or may have evolved for one function to later be co-opted for another. To test this, we looked for correlations of bare part size, degree of bare part size dimorphism, and erectile capacity (ability to very rapidly enlarge bare parts) with variables that might reflect either sexual selection or natural selection (table 1). If bare parts are present due to sexual selection, we should find strong correlations between bare parts and traits such as dimorphism in body size, plumage colour or ornamentation, mating system or type of parental care (e.g. biparental or female only). By contrast, if bare parts reflect natural selection via thermoregulation, we should find strong correlations with variables such as temperature, altitude, solar radiation, or vegetative cover for both sexes. We also tested the above hypotheses in Phasianidae, the largest family in Galliformes, accounting for over 60% of species in the order. Strong sexual selection has primarily been suggested in Phasianidae, which includes species with extravagant feather ornaments (e.g. pheasants, peafowl and monals) and species with lek mating and intra-sexual fights (e.g. grouse and junglefowl). On the other hand, Phasianidae also includes species that have little plumage elaboration or no bare parts (e.g. partridges and the Old World quail). Thus, there is a substantial variation within Phasianidae to assess correlation with bare parts. Finally, we conducted ancestral trait reconstructions to estimate the ancestral state and state transition of bare part traits to better understand how bare part traits have changed through time.

**Table 1.** Hypotheses and predictions for potential correlates of bare part traits.

hypothesis	bare part trait	prediction	predictor	justification
***heat dissipation*** bare parts enable birds to avoid hyperthermia by increasing surface area for loss of body heat to the environment.	**bare part size** (both male and female)	larger in hotter and more humid places	temperature	species in warmer environments will need more bare area to dissipate excess body heat
			NDVI	higher humidity reduces efficacy of evaporation (panting) for heat dissipation, so larger bare parts needed for convective heat loss (assumes more vegetation in humid places)
			solar radiation	solar radiation heats bare parts, removing the thermal gradient needed for heat dissipation, so smaller bare parts are needed
			altitude	air movement (wind) necessary for convective heat loss is less at lower altitudes, so larger bare parts are needed to dissipate heat
	**size change**	greater in hotter and more humid places	the same as for bare part size (above)	courtship display generates extra body heat, creating a greater demand for surface area for heat loss in males during courtship
	**sexual size dimorphism**	less in hotter and more humid places	temperature NDVI	females will share same risk of hyperthermia as males and will need same amount of bare area as males
			solar radiation Altitude	females will share same risk of hyperthermia as males and will need same amount of bare area as males
***sexual selection*** increased intrasexual (male-male) competition and/or intersexual selection (female choice) should select for large bare parts and greater difference in bare parts between two sexes.	**bare part size** (male)	larger with stronger sexual selection	mating system	greater variance in male mating success in polygyny places greater selective pressure on male traits
			parental care	stronger selection on male indicators of good genes if that is what female favours
			sexual plumage dimorphism	dimorphism is associated with strength of sexual selection
			sexual size dimorphism	body size dimorphism suggests intrasexual competition (e.g. male combat success)
	**size change**	greater with stronger sexual selection	mating system	same as above for bare part size (male)
			parental care	
			sexual plumage dimorphism	
			sexual size dimorphism	
	**sexual size dimorphism**	greater with stronger sexual selection	mating system	same as above for bare part size (male)
			parental care	
			sexual plumage dimorphism	
			sexual size dimorphism	

## 2. Methods

### 2.1. Phylogeny for Galliformes

We used the phylogeny from Kimball *et al*. [33] which consisted of 264 species of Galliformes (88% of the order, and at least 85% of each family): 160 species within Phasianidae and 104 species outside Phasianidae, based on the IOC taxonomy v10.1 [34]. This phylogeny was transformed into an ultrametric tree for subsequent comparative analyses and trait reconstruction using the ‘chronos’ function from the package *ape* [35] in R [36] with correlated substitution rate variation and *λ* = 1. We also subset the Galliformes tree to only include the 160 species in Phasianidae for taxon-specific analyses using the ‘drop.tip’ function of *ape* with internal trimming. R scripts are available in https://github.com/balaenazhao/GalliformesBareParts.

### 2.2. Scoring bare parts

We scored male and female bare parts for each species included in the phylogeny (electronic supplementary material, data S1). The scoring system was developed by E.A.H. in consultation with other co-authors and E.A.H. assigned all scores (with some discussion by other co-authors). For species containing multiple morphs or subspecies, we scored the most common form. We used description and illustration from *Birds of the World* [37] and, when images on the site were not clear, additional images and videos on the web, to score bare parts. Here, bare parts are defined as visibly featherless areas on the head, neck, and breast. They included hard protrusions (e.g. casques and knobs); soft, vascularized protrusions (e.g. wattles, dewlaps, lappets, combs, caruncles, and snoods); inflatable structures (e.g. gular sacs); and any visibly exposed skin, including areas where feathers are present but thin (e.g. ceres, lores, and throat patches) or where feathers are erected to reveal otherwise hidden bare patches (e.g. throat patches of some grouse species).

Bare part size was scored on a scale from 0–5 (figure 1*a*) for both sexes in the flaccid, unerected state: 0 indicating the total absence of bare parts; 1 indicating a small area of exposed skin (most often ceres, bare supraorbital patches, or small dewlaps/throat patches); 2 indicating the presence of an area of bare skin such as a lore or a small but protruding brow comb or gular dewlap; 3 indicating the presence of a moderate amount of bare skin (e.g. entire head or entire neck) without protrusions or the presence of a moderately sized protrusion such as a comb or wattle; 4 indicating the presence of a larger protrusion or some combination of bare skin and a moderately sized protrusion; and 5 indicating the presence of multiple large fleshy protrusions or a large continuous bare area that covers the entire head and neck such as in the Wild Turkey. Sexual dimorphism of bare parts was scored by subtracting female bare part size score from male bare part size score.

We scored bare part size change by estimating the change in male bare part surface area between the flaccid state and the display state when structures are at their largest during courtship (e.g. expanded tragopan lappets [figure 1*b*], erected ptarmigan brow combs and inflated grouse gular sacs). Thus, each size change score is a multiplier for the size score of the structure in its flaccid state and represents an ‘*X*-fold’ change in size. Therefore, taxa that lack bare parts for this variable received a score of zero, and a score of 1x represented the presence of a bare part that does not change size. Any score above 1 reflects an increase in size, where 1.5× would mean a structure changes size by 1.5 times and 2× would mean a structure doubles in size, and so on. These scores were not restricted to a ranked scale, as we wanted to accurately represent the variation in this trait across Galliformes.

### 2.3. Validation of bare part scores

We validated our scores by comparing subsets of them with datasets from two independent studies (electronic supplementary material, data S2). First, we extracted the rank scores of male bare parts from Buchholz [32] and reconciled the species names to match our samples. Buchholz [32] used a similar approach to what was applied in our study and scored bare part flaccid size from zero to five but using different images and did not consider casques such as those present in some Cracidae species. Therefore, we performed Spearman correlation tests for two datasets: 1) all reconciled taxa (*N* = 234); and 2) all taxa excluding Cracidae (*N* = 192). Secondly, we compared our scores of male bare part size with the measurements of some Phasianidae species [38]. Smith [38] used ImageJ [39] to measure the pixel number of bare part area and the entire body region shown on lateral photos and calculated the bare part to body ratio as the score of bare part size. The measurements were based on lateral views of the birds, thus could not capture the entire frontal bare parts if present, such as the throat pouch in resting male tragopans (figure 1*b*). We performed Spearman correlation tests for: 1) all reconciled taxa (*N* = 34); and 2) all taxa excluding *Tragopan* spp. (*N* = 30).

### 2.4. Putative sexually selected traits

We selected a suite of life history and environmental variables that could potentially influence bare parts in different ways (table 1). All the traits with respective references can be found in electronic supplementary material, data S3.

#### 2.4.1. Sexual dimorphism in plumage and ornamentation

Across Galliformes, females have been shown to prefer more highly ornamented males [40,41], indicating plumage may be a target for sexual selection across Galliformes. We scored plumage differences between the sexes, rather than scoring plumage coloration *per se,* because our focus was to assess putative opportunities for sexual selection as an indication of the strength of sexual selection in each species. Using *Birds of the World* [37] and, when images were not clear or to verify information, images on the web, we scored seven characters. For four body regions: 1) head and neck, 2) back of body, 3) breast and belly, and 4) wing and side of body, species were scored as 0 (no obvious differences), 1 (slight differences, such as males slightly darker), or 2 (sexes obviously different). In addition, we looked for sexual dimorphism for three specialized plumage categories: 5) tail, 6) crest and 7) other, which included specialized plumage structures such as the elongated secondaries of argus pheasants. For these structures, species were scored either as 0 (identical or very similar between the sexes), or 1 (very different, such as present in males and absent in females). We then summed up the scores of the above seven characters as the score for plumage colour and ornament sexual dimorphism (ranging from 0–11; hereafter referred to as ‘plumage dimorphism’).

#### 2.4.2. Body size dimorphism

Larger male size is preferred by females of some Galliformes [13,42], so size dimorphism may reflect sexual selection. Male and female body mass data were gathered from *Birds of the World* [37] and the *CRC Handbook of Avian Body Masses* [43]. For species with missing information from either of these sources, VERTNET [44] was searched. A minimum of one individual per sex per species was considered necessary for inclusion and the average mass was taken when there were multiple specimens. For species missing information from any of these three sources, Google Scholar was searched as of May 2020. Body size sexual dimorphism was calculated as the female mass subtracted from the male mass divided by the female mass.

#### 2.4.3. Mating system and parental care

We assessed systems of reproductive behaviour since these traits are good indicators of the strength of sexual selection acting on a species [45–47]. *Birds of the World* [37] and Cockburn's publication on avian parental care [48] were used in identifying mating systems and parental care. Mating system was classified into the occurrence versus the absence of monogamy to simplify cases in which species practise multiple mating strategies [37], such as the Common Quail (*Coturnix coturnix*) and the Japanese Quail (*C. japonica*). Post-hatching parental care was categorized into discrete numerical scores of mound-building (0), uniparental (1; male or female parent alone performs majority of parental care), and biparental (2; both male and female parents contributing to parental care). For species missing information from any of these three sources, Google Scholar was searched as of May 2020.

### 2.5. Environmental data collection

To obtain accurate estimates of environmental data, we downloaded occurrence data from GBIF (https://www.gbif.org) for each Galliformes species represented in our phylogeny and performed the following data curation steps in R. We removed duplicate and overlapping occurrence points for each species using *raster* [49] to ensure that multiple occurrence points less than 0.0083° (approx. 900 m) apart were condensed into one occurrence point to reduce sampling bias across geographical localities that may vary in terms of accessibility. We also removed occurrence points that fall out of the expected species distribution (with a buffer of 0.9°, i.e. approximately 100 km) based on the range maps downloaded from BirdLife International [50] using R packages *rgdal* [51], *sf* [52] and *sp* [53], to account for reports of potential captive or misidentified birds.

We assessed the following environmental variables that might predict thermoregulatory capacity: altitude (median, 25% and 75% quartiles); temperature annual mean, minimum (coldest month) and maximum (hottest month); solar radiation annual mean, minimum (cloudiest month) and maximum (sunniest month); and normalized difference vegetation index (NDVI) annual mean, minimum (lowest vegetation month), and maximum (highest vegetation month). Altitude data (30′ resolution) were downloaded from Global Multi-resolution Terrain Elevation Data 2010 [54], temperature data (30′ resolution) from CHELSA (climatologies at high resolution for the Earth's land surface) [55], NDVI data from the USGS EROS archive as Advanced Very High Resolution Radiometer (AVHRR) [56] and solar radiation data downloaded from WorldClim [57]. Environmental variables were extracted for the occurrences using *raster* [49]. The median of each variable across all occurrence points per each species was calculated (electronic supplementary material, data S4).

Two species of Galliformes are considered migratory: the Common Quail and the Japanese Quail [37]. Location and thus environmental conditions vary depending on the time of year; frigid temperatures and complete vegetation loss are observed during winter in their breeding grounds. Breeding season was classified as April through October, months when both species were observed in their breeding ranges according to ebird.org occurrence points [58]. The non-breeding season was classified as November through March for both species. The minimum was taken between the temperature of the coldest month of the year-round resident range and the coldest month of the non-breeding range between November and March. Likewise, the maximum was taken between the temperature of the hottest month of the year-round resident range and the hottest month of the breeding range between April and October. The mean annual temperature was taken from the year-round resident range for each species. The same procedure was completed with solar radiation and NDVI. Altitude data from the breeding, non-breeding, and resident ranges were combined, and the median, 25% quartile and 75% quartiles were calculated.

For the Niuafoou Scrubfowl *Megapodius pritchardii*, the species range according to BirdLife International (2020) is just on Niuafo'ou, Tonga, and this range is smaller than that of the NDVI raster resolution. However, there were GBIF occurrences of this species on neighbouring islands to Niuafo'ou. Therefore, for the Niuafoou Scrubfowl only, all occurrence points, including those that did not overlap with the BirdLife International species range, were used to extract NDVI information.

To reduce the number of environmental variables, we performed principal component analyses (PCA) using the prcomp function for each type of environmental variable and retained the first principal component: altitude PC1, temperature PC1, solar radiation PC1, and NDVI PC1. Since temperature PC1 was negatively correlated with the original variables (electronic supplementary material, data S5), we multiplied each data point by –1 in all subsequent comparative analyses to facilitate easy comparison.

### 2.6. Ancestral state estimation and phylogenetic signal

To understand the ancestral states of bare parts across Galliformes and examine the potential transitions through the evolutionary history, we reconstructed ancestral states of all bare part traits on the Galliformes phylogeny. We used stochastic character mapping [59] for trait reconstruction to accommodate uncertainty in ancestral state estimation. Since our characters were coded as discrete (either numeric discrete or categorical), we used SIMMAP [60] to simulate stochastic trait evolution histories on the tree and assumed unordered states. For bare part size change, we used ‘0’ to denote no bare part, ‘1’ for bare part present but no size change and other scores for bare part size fold change. The equal rates, symmetric rates, and all rates different models were tested for each trait for one iteration, and the likelihood ratio test was subsequently used to identify the best fitting model. We then used the chosen model to perform and map 100 simulations of trait reconstruction on the phylogeny using the ‘make.simmap’ function in the R package *phytools* [61]. We summarized the number of ‘increase’ (lower score to higher score) or ‘decrease’ (higher to lower) based on the average number of changes between state.

Prior to comparative analyses, we addressed the issue of missing character data. Missing data for mating system (6.1% of total taxa) and body size dimorphism (3.8%) were estimated using ancestral reconstruction in *phytools* for discrete traits (mating system) using the functions ‘make.simmap’ and for continuous traits (body size dimorphism) using ‘anc.ML’. The discrete character state function outputs the probabilities of each state (monogamous and non-monogamous) for each tip and node according to 100 simulated character state transformations. For each missing tip for mating system data, the character state with the higher probability was designated as the missing tip state. The continuous character state function was run under the Brownian Motion model to estimate missing tip values for body size sexual dimorphism. The function was run with enough iterations (100) to ensure that the model converged.

### 2.7. Phylogenetic comparative analyses

To examine the effects of the eight predictor variables (altitude, NDVI, solar radiation, temperature, mating system, parental care, sexual plumage dimorphism and sexual body size dimorphism) on a single response variable, we ran phylogenetic generalized least squares (PGLS) for each response variable (male size, female size, sexual size dimorphism and male size change) in Galliformes and Phasianidae using the ‘gls’ function of the R package *nlme* [62]. For male size change, we excluded the species that had no bare parts from the phylogeny and data matrix, which resulted in a dataset containing 185 species across Galliformes, and a Phasianidae only dataset containing 105 species. The OU model was consistently the best-fitting model in terms of log-likelihood scores and AIC scores when compared to the Brownian Motion and Pagel's lambda models. For each of the four bare part response variables, we tested all possible combinations of eight predictors (*N* = 255 models). To evaluate the relative importance of the predictors for each bare part trait, we first discarded models that had a higher AIC score than the best AIC model by at least 2 [63] and calculated the Akaike weights for the remaining candidate models [64]. We summed the Akaike weights for each candidate model that included this predictor [63]. The higher the summed weight is, the more frequent a predictor appears in the top-ranked models. We also standardized the predictors (except for mating system) by centering and dividing by one standard deviation and ran a PGLS using the full model for each bare part trait. We then used R package *dotwhisker* [65] to plot out the effect size of each predictor for the full model.

### 2.8. Exploring the effects of sage-grouse

We noted that two sage-grouse species (*Centrocercus* spp.) were potential outliers in terms of the ability to change the size of bare parts as their gular sacs can be inflated dramatically with air during display (see Results). Therefore, we removed the two sage-grouse from our data matrix as well as from the associated phylogeny and repeated the above comparative analyses to examine how these extreme taxa may influence the correlations between bare part and predictor variables.

## 3. Results

### 3.1. Validation of bare part scores

In comparing our rank scores with data using a similar ranking system [32], the rank scores were highly correlated with our scores (all reconciled taxa, correlation coefficient rho = 0.837; and all taxa excluding Cracidae, rho = 0.881) with *p* values both less than 2.2 × 10^−16^. When compared to the study using two-dimensional measurements [38], the values were lower (all reconciled taxa, rho = 0.697, *p* = 4.70 × 10^−06^), though these improved with removal of tragopans which have some bare parts mostly visible using the frontal view (all taxa excluding *Tragopan* spp., rho = 0.730, *p* = 4.63 × 10^−06^). This indicates an influence of focusing on the lateral view when structures may be most visible with a frontal view. Therefore, we feel using rank scores allowed for a more comprehensive estimate of the overall complex three-dimensional structures. Even when assessed independently by different people and using different bird images, the rank scores were very similar.

### 3.2. Variation in bare parts across Galliformes

**Figure 3.**
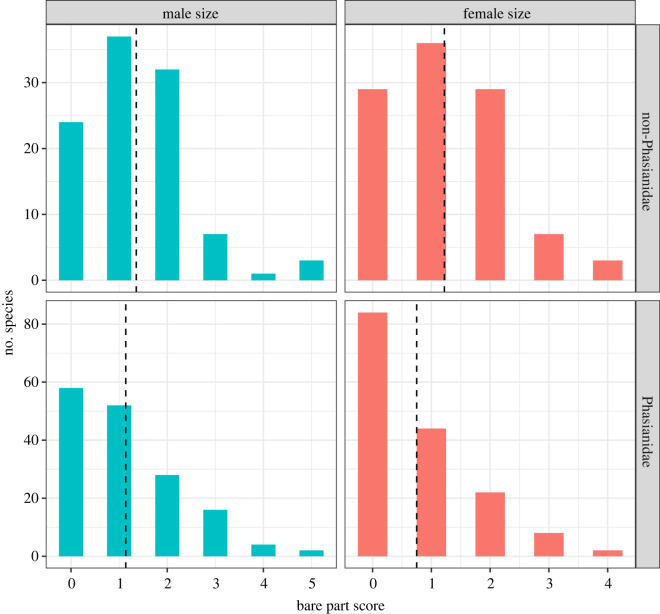
Bare part size of male (teal) and female (red) in non-Phasianidae (above) and Phasianidae (below). Dashed line denotes the mean value for each data set.

Overall, there was variation in bare parts and their distribution among all five galliform families (figure 2). However, species within Phasianidae had higher variation in most bare part traits when compared to the other four families. Bare parts were more common on females in the four non-Phasianidae families, and in those families, there was less sexual dimorphism in bare parts as compared with Phasianidae. On average, both sexes of non-Phasianidae species had larger bare parts than those of Phasianidae, and Phasianidae a greater proportion of species with no bare parts (figure 3).

Estimation of ancestral traits can give us a glimpse into the evolutionary trajectory of bare parts and how labile these traits are across the Galliformes tree. Stochastic mapping showed the ancestral states for Galliformes were much less resolved (low probabilities of the most probable states) than those for Phasianidae (electronic supplementary material, data S6). The most recent common ancestor (MRCA) for Phasianidae was likely to be sexually monomorphic and lacking bare parts. Based on the current phylogeny, erectile bare parts evolved several times independently within Phasianidae, and once in Megapodiidae (figure 2). Since the ancestral state of male size change for MRCA of Phasianidae was most likely to be zero, i.e. no bare part present, the ability to change bare part size likely evolved late in the evolutionary history. Overall, there appeared to be a large number of transitions of bare part character states across Galliformes. For bare part sizes, increases were more likely to occur than decreases in both sexes, especially for females.

### 3.3. The strongest predictors for bare parts vary across clades

**Figure 4.**
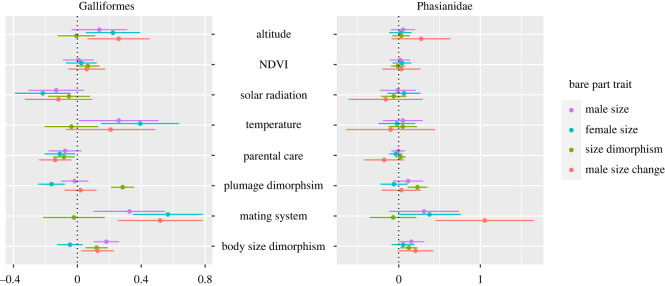
Effect size of each predictor predicting a bare part trait in the full PGLS model for Galliformes and Phasianidae. For mating system, not being monogamous is important.

Using PGLS, we found a combination of both environmental and sexually selected traits that strongly predicted variation in bare part sizes of both males and females (figure 4; table 2; full model results can be found in electronic supplementary material, data S7). Across Galliformes, bare parts were larger for both sexes in non-monogamous species, and species that occur in regions with overall hotter and less sunny environments. Parental care and plumage dimorphism were found to be especially important for predicting female size, and body size dimorphism for male size. When examining the effects of different predictors on bare parts in Phasianidae alone, none of the environmental variables were found important for predicting male nor female size, while all bare part traits were associated strongly with at least one putative sexually selected trait.

**Table 2.** Assessing the relative importance of predictors for four bare part variables in Galliformes and Phasianidae based on results from PGLS using complete dataset. Relative importance of each predictor is the summed AIC weights of candidate models in which this predictor appears. For mating system, not being monogamous is important.

	**proxy for sexual selection**				**proxy for natural selection**			
	mating system	parental care	plumage dimorphism	body size dimorphism	NDVI	altitude	solar radiation	temperature
Galliformes								
male size	1.000	0.587	0.654	1.000	0.218	0.450	1.000	1.000
female size	1.000	1.000	1.000	0.430	0.304	0.584	1.000	1.000
size dimorphism	0.212	1.000	1.000	1.000	0.619	0.078	0.180	0.288
male size change	0.811	0.382	0.158	0.117	0.137	0.577	0.000	0.175
Phasianidae								
male size	1.000	0.089	0.096	0.837	0.097	0.100	0.123	0.090
female size	1.000	0.093	0.176	0.134	0.103	0.095	0.084	0.085
size dimorphism	0.126	0.125	1.000	1.000	0.110	0.112	0.112	0.114
male size change	1.000	0.087	0.709	0.229	0.077	0.077	0.076	0.075

### 3.4. Evaluating the effects of sage-grouse

**Figure 5.**
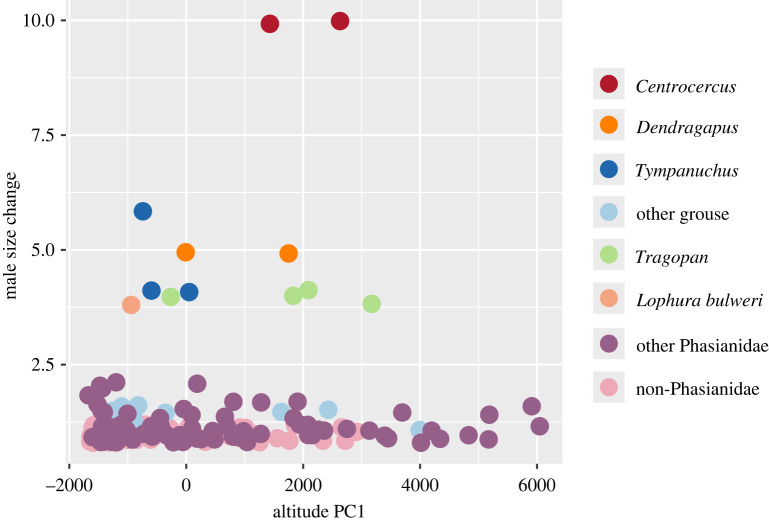
Scatter plots for Galliformes using altitude (PC1) as X-axis and male bare part size change score as Y-axis. A point jitter of 0.2 was created for the Y-axis. Taxa without bare parts have been excluded from this graph. Taxa with exceptionally high score for male size change are colour-coded based on genus name. ‘Other grouse’ include taxa from *Bonasa*, *Tetrastes*, *Falcipennis*, *Tetrao*, *Lyrurus* and *Lagopus*.

We noticed that mating system and altitude were likely important in predicting capacity to inflate or erect bare parts in males across Galliformes, whereas in Phasianidae, only mating system and plumage dimorphism were found to be very important (electronic supplementary material, data S7). This is puzzling since the ability to change bare part size was only scored in Phasianidae (all restricted in the ‘erectile clade’, Kimball *et al*. [3]) and one Megapodiidae species (Australian Brushturkey *Alectura lathami*). These motivated us to examine the relationships between bare part size change and altitude (figure 5). Two data points with the largest size change, the two sage-grouse (*Centrocercus* spp.), were so distinct we considered whether these could have driven the correlation and may have had a major impact on the results regarding size change.

After removing the two sage-grouse species, we still found both environmental and putative sexually selected traits predicted male and female bare part size in Galliformes (electronic supplementary material, figure S2; data S8). By contrast, when just considering Phasianidae, none of the environmental variables were found to be important for predicting bare parts. The most important predictors for bare part sizes in Phasianidae were mating system, plumage dimorphism and body size sexual dimorphism, all putative sexually selected traits. For size change, the importance of mating system and altitude dropped significantly for Galliformes after removing the sage-grouse, whereas for Phasianidae, the most important predictors for size change were still mating system and plumage dimorphism.

## 4. Discussion

We tested two non-mutually exclusive hypotheses to understand the selective pressures that underlie the specialized bare parts in Galliformes, that of thermoregulation (natural selection) and sexual signalling (sexual selection). We found evidence of both sexual and natural selection driving bare part evolution across all Galliformes. When restricted to Phasianidae, traits that may reflect sexual selection were the primary predictors of bare parts. Our results suggest that bare parts first evolved for multiple functions including both heat transfer with the environment and signalling, but within Phasianidae primarily function as sexual signalling structures. Our results also demonstrate how a few taxa (less than 1% for the two sage-grouse) may influence results and that exploring the data can be useful to understand whether a few taxa may have a disproportionate impact on overall conclusions.

### 4.1. Bare parts play a thermoregulatory role outside Phasianidae

Many studies have suggested thermoregulatory function of bare parts for birds living in hot climates, such as ratites [12,66], storks [20], caracara and vultures [1]. Across Galliformes, we found that both sexes tend to have larger bare parts in hotter but less sunny environments. Our results support the thermoregulation hypothesis that bare parts may expel excess heat in hotter or more humid conditions and highlight that the needs for thermoregulation are essential for both males and females. Although temperature and solar radiation are usually positively correlated, our results consistently suggested antagonistic effects of temperature and solar radiation on the bare part size of both sexes across Galliformes. We also found that non-Phasianidae birds, on average, have larger bare parts in both sexes than Phasianidae (figure 3). In tropical environments where many non-Phasianidae species occur, low solar radiation and high temperature such as in dense rain forests can lead to high humidity, which is known to decrease the efficiency of evapotranspiration [67]. Pollock *et al*. [68] found that tropical birds had significantly lower heat tolerance limits on average than temperate birds. Thus, more exposed skin might be important for tropical birds to dissipate excess heat. In addition, birds living in moist environments can afford to lose more water via evapotranspiration. Exposed skin, which increases the efficiency of evapotranspiration, should be smaller or absent in arid climates where water availability is much scarcer. Supporting this, Crowe [69] found that bare parts are smaller for populations in drier locations for the polytypic Helmeted Guineafowl *Numida meleagris*.

Bare parts could also increase the efficiency of heat absorption, which could be beneficial for taxa in cold environments. For example, a recent study of the Helmeted Guineafowl found that the bare throat sac of the black-throated subspecies is often larger during winter months probably to maximize heat absorption via solar radiation [70]. However, across Galliformes, we found that species that occur in cold but sunny regions tend to have small bare parts, suggesting that heat absorption from solar radiation may not offset the heat loss through bare skin in cold environments therefore is less effective than insulation from layers of feathers. On the other hand, bare part coloration can also contribute to thermoregulation, therefore, a large bare part may not always be necessary in cold environments if the bare parts are darkly pigmented, such as suggested in the Helmeted Guineafowl [70], although most often dark plumage might be a more effective thermal adaptation for birds inhabiting such environments [71]. In areas with high solar radiation, bare parts may be prone to UV damages, therefore, UV radiation may also pose selection on smaller bare parts or darkly pigmented skin (e.g. [72]).

### 4.2. Bare parts under sexual selection

We uncovered strong evidence that sexual selection likely plays a role in the evolution of bare parts across Galliformes, especially in Phasianidae. Across Galliformes, we found that variation in the traits known to be sexually selected significantly predicted bare part size in both males and females. Studies on sexual selection in several galliform species suggest both inter- and intrasexual selection for bare parts in males. In the Wild Turkey [13] and Red Junglefowl [73], females prefer to mate with males with larger bare parts, while in the Black Grouse, comb size was found to be positively correlated with copulatory success [15]. Sexual selection may favour multiple male signals simultaneously during female mate choice [74–77], which could explain the strong correlations we observed between bare part and plumage/body size sexual dimorphism (though some have suggested plumage traits may not be important in sexual selection in Galliformes [7,78]). However, in several species there is no significant female preference for male bare part size, such as in the Ring-necked Pheasant *Phasianus colchicus* [79], the Rock Ptarmigan *Lagopus muta* [80], and the Sharp-tailed Grouse *Tympanuchus phasianellus* [81]. Whereas in the Lesser Prairie-chicken *Tympanuchus pallidicinctus*, successful males tend to have smaller combs but with brighter and more saturated colours [74]. Unfortunately, for the majority of species, there is no data on whether or not females use bare parts in mating decisions.

For intrasexual competition, the size of bare parts often correlates with male aggression and intrasexual competition success (likely mediated by testosterone), such as in the ptarmigans [80,82] and Red Junglefowl [25]. Body size [83] may be another strong correlate of intra- versus intersexual signalling. We found that body size dimorphism was positively correlated with male bare part size, i.e. males that have large bare parts also tend to have larger body size than intraspecific females. Females may choose males that win male–male competitions or have high aggression [74,84–86] and in effect indirectly select for bare parts even if there is no direct female preference for the trait itself. For example, females of the Rock Ptarmigan choose males according to bare part abrasion and damage rather than size, potentially as a proxy of male dominance and competitive ability [80]. However, the ability to rapidly enlarge bare part is often found in non-monogamous species, such as those with lekking behaviour and intra-sexual fights. In addition, we also found stronger correlation when considering body size dimorphism than parental care. These together may indicate that bare parts play a stronger role in male–male competition than female mate choice, although teasing apart intra- versus intersexual selection for bare parts is challenging.

Female bare parts could be maintained by male mate choice and/or female intrasexual competition for males and other resources important to reproduction. Even in polygynous species such as the Red Junglefowl, males invest more sperm in females with larger combs, a potential signal for higher fecundity [87–89]. Alternatively, females may sometimes express traits that are under strong selection in males [90,91], and that may also contribute to the presence of bare parts in females—especially in species where thermoregulation (particularly heat loss) may not be critical, as is true for many phasianids.

### 4.3. Interplay between sexual and natural selection on bare parts

Our results suggest that bare parts evolved in Galliformes for both thermoregulation and sexual signalling but are primarily under strong sexual selection within the Phasianidae. These are consistent with the previous hypothesis that bare parts evolved for thermoregulation and subsequently emerged as important signalling structures [32]. Many putative sexually selected traits in birds are likely subject to both sexual and natural selection, for example, plumage coloration (reviewed by [92]), body size (e.g. [93]), and tail streamers (e.g. [94]). Evolutionary changes in trait function could be due to a changed balance between sexual and natural selection, such as when species colonize a novel environment [95]. Outside of the Phasianidae, most extant Galliformes occur in the tropics, where the duration of breeding season might be extended due to high food availability throughout the year. Competition for ecological resources can impose selective pressure on both sexes for ornamentation as informative signals [96,97]. Therefore, bare parts may be under selection for both thermoregulatory and reproductive purposes in many non-phasianid species. Phasianidae, on the other hand, has successfully colonized the temperate (even subarctic) zone and rapidly diversified in the montane regions, such as in the Himalayas [98], where selective pressure favouring structures that promote heat dissipation should be relaxed. In colder regions, bare parts may lead to excessive heat loss and should be selected against. Erectile bare parts theoretically could benefit species living in cold climates since this feature conceals exposed skin outside the periods of display. However, we did not observe a strong correlation between environmental variables and male size change for Phasianidae, though we did for Galliformes as a whole. Nevertheless, our findings showcased how a trait may have evolved for one function but was later shifted for another and highlighted how putative sexually selected traits may also exhibit evolutionary change driven by natural selection.

## Data Availability

Data and relevant code for this research work are stored in GitHub: https://github.com/balaenazhao/GalliformesBareParts/ and have been archived within the Zenodo repository: https://doi.org/10.5281/zenodo.10302846 [99]. Supplementary material is available online [100].
